# Association of pregnancies with risk of multiple sclerosis

**DOI:** 10.1177/13524585221080542

**Published:** 2022-03-18

**Authors:** Christiane Gasperi, Alexander Hapfelmeier, Antonius Schneider, Klaus A Kuhn, Ewan Donnachie, Bernhard Hemmer

**Affiliations:** Department of Neurology, Klinikum rechts der Isar, TUM School of Medicine, Technical University of Munich, Munich, Germany; Institute for AI and Informatics in Medicine, TUM School of Medicine, Technical University of Munich, Munich, Germany/Institute of General Practice and Health Services Research, TUM School of Medicine, Technical University Munich, Munich, Germany; Institute of General Practice and Health Services Research, TUM School of Medicine, Technical University Munich, Munich, Germany; Institute for AI and Informatics in Medicine, TUM School of Medicine, Technical University of Munich, Munich, Germany; Bavarian Association of Statutory Health Insurance Physicians, Munich, Germany; Department of Neurology, Klinikum rechts der Isar, TUM School of Medicine, Technical University of Munich, Munich, Germany/ Munich Cluster for Systems Neurology (SyNergy), Munich, Germany

**Keywords:** Multiple sclerosis, case–control studies, pregnancy, autoimmune diseases, risk factors, health services research

## Abstract

**Background:**

Pregnancies have an impact on the disease course of multiple sclerosis (MS), but their relationship with MS risk is yet unclear.

**Objective:**

To determine the relationships of pregnancies and gynecological diagnoses with MS risk.

**Methods:**

In this retrospective case–control study, we assessed differences in gynecological International Classification of Diseases, 10th Revision (ICD-10) code recording rates between women with MS (*n* = 5720), Crohn’s disease (*n* = 6280), or psoriasis (*n* = 40,555) and women without these autoimmune diseases (*n* = 26,729) in the 5 years before diagnosis.

**Results:**

Twenty-eight ICD-10 codes were recorded less frequently for women with MS as compared to women without autoimmune disease, 18 of which are pregnancy-related. After adjustment for pregnancies, all codes unrelated to pregnancies were still negatively associated with MS. In a sensitivity analysis excluding women with evidence for possible demyelinating events before diagnosis, all associations were more pronounced. In comparison to women with psoriasis, most associations could be confirmed; that was not true in comparison to women with Crohn’s disease.

**Conclusion:**

Our findings provide evidence for a possible protective effect of pregnancies on MS risk likely independent of or in addition to a previously suggested reversed causality. The negative associations of gynecological disorders with disease risk need further investigation. The associations might be shared by different autoimmune diseases.

## Introduction

Women are affected by multiple sclerosis (MS) more often than men and the sex difference in prevalence rates further increased during the last decades.^1–4^ The reasons for the higher MS prevalence in women are uncertain, but genetic and hormonal factors have been implicated.^5^ Pregnancies have a known impact on the MS disease course as relapse rates decrease substantially during pregnancy.^6,7^ Whether pregnancies also have an impact on MS risk is not yet clear. Some studies reported negative associations of having children with MS risk in women but not in men, which argues for a biological impact of pregnancies on MS risk.^8,9^ Other studies, however, found that women as well as men had a lower risk of developing MS when being parents.^10,11^ In addition, in these studies, the negative relationship of parenthood and MS could only be observed for periods of 5 or 10 years before diagnosis. These results led to the hypothesis of a possible reversed causality, that is, a lower reproductive activity or ability in patients with already ongoing MS even years before diagnosis.^10,11^

In this retrospective case–control study, we investigated the recording rates of gynecological International Classification of Diseases 10th Revision (ICD-10) codes and ICD-10 codes related to reproductive medicine in women with MS in Southern Germany in the 5 years before first diagnosis. We used ambulatory claims data held by the Bavarian Association of Statutory Health Insurance Physicians (BASHIP). The primary aim was to investigate differences in ICD-10 code recording rates for women with MS as compared to controls to get an insight into the relationship between pregnancies and MS risk. To assess whether the observed associations are specific for MS, we used two additional control cohorts of women newly diagnosed with Crohn’s disease (CD) or psoriasis.

## Materials and methods

### Data

Anonymous ambulatory claims data from 2005 to 2017 from all members of the statutory health insurance in the German federal state of Bavaria were used. According to the Guidelines and Recommendations for Good Practice of Secondary Data Analysis^12^ approval by an ethical standards committee on human experimentation or written informed consent from the participants were not needed. Approval was, however, obtained from the data protection officers of the BASHIP.

We defined a cohort of women newly diagnosed with MS and three control cohorts of women with CD, with psoriasis and women without any of these three autoimmune diseases (AIDs). Except for the last cohort, two recorded first secured ICD-10 codes of the respective disease (G35 for MS, K50 for CD, and L40 for psoriasis) in two separate billing quarters between 2010 and 2017 were required. All women with MS further had to have had at least one neurologist visit. Women with more than one of the three AIDs and women with secondary progressive MS as the first recorded diagnosis were excluded. We further removed women with recordings of other possible demyelinating or inflammatory diseases of the central nervous system in the 5 years before diagnosis (Supplementary Table 1 shows the ICD-10 codes used for this restriction). The control cohort without any of the AIDs was matched to the MS cohort in a 5:1 ratio by age and district of residence, assigning each individual the quarter of first diagnosis from their matching partner. We selected women with age at diagnosis between 21 and 50 years.

In a previous study, we observed higher recording rates for 43 ICD-10 codes for patients with MS as compared to controls in the 5 years before diagnosis.^13^ Many of these are neurological or neurovascular ICD-10 codes or correspond to symptoms that could represent demyelinating events. We, therefore, performed a sensitivity analysis where we removed women with recorded neurological or neurovascular ICD-10 codes or codes suggestive of demyelinating events and associated with MS in our previous study^13^ in the 5 years before diagnosis (Supplementary Table 1).

### Statistical analysis

In the main analysis, we investigated the recording rates of ICD-10 codes related to gynecological symptoms and diseases (all female-specific codes) and codes related to reproductive medicine (Supplementary Table 2) recorded in at least 0.5% of all women in the 5 years prior to diagnosis in women with MS as compared to the cohort without AID. We excluded the last quarter before diagnosis. We created binary predictor variables indicating whether a code was recorded at least once (yes) or never (no). We investigated the associations of these predictor variables with MS diagnosis by means of unconditional logistic regression and included age at diagnosis (categories 21–25 years, . . ., 46–50 years) to obtain adjusted effect sizes.^14^ In cases of complete or quasi-complete separation, we used Firth’s biased-reduced logistic regression.^15,16^

For ICD-10 codes associated with MS in the main analysis, we performed a sensitivity analysis for which we excluded women with evidence for a possible demyelinating event in the 5 years before first diagnosis, analyses for each of the 5 years separately (excluding the last quarter before diagnosis), and an analysis adjusting for pregnancies. As the data do not contain a specific ICD-10 code for pregnancy we used the recordings of pregnancy-related ICD-10 codes to identify women with at least one versus no pregnancies in the 5 years before diagnosis.

The significant findings were further analyzed in comparisons of the MS cohort to the two cohorts of women with CD or psoriasis. We further calculated the frequency of gynecologist encounters in the 5 years before diagnosis.

To investigate a possible dose effect of pregnancies on MS diagnosis, we estimated the number of pregnancies by counting the number of recordings of pregnancy-related ICD-10 codes that were at least 12 months apart. We calculated odds ratios (ORs) of MS diagnosis for women with one versus zero, two versus zero and ⩾ three versus zero pregnancies using the cohort of women without AID as controls.

We corrected for multiple testing using Sidak’s correction to control the familywise error rate at a 5% significance level. In the main analysis, the number of tests was 77; in the sensitivity analysis and the analysis adjusted for pregnancies, 28 and 10 ICD-10 codes were analyzed, respectively. We computed all analyses with R3.6.1 (The R Foundation for Statistical Computing, Vienna, Austria).

### Data availability

The open distribution of the data is prohibited by the data protection regulations effective in Bavaria. Researchers may contact the BASHIP or the corresponding author to request data access.

## Results

### Study cohorts

The study cohorts consisted of 5720 women newly diagnosed with MS; 40,555 women without any of the AIDs; and 26,729 and 6280 women newly diagnosed with psoriasis or CD, respectively (Table 1).

**Table 1.** Descriptive statistics of the study cohorts.

Analysis	Cohort	Number of women	Distinct ICD-10 codes (median, interquartile range)	Age at first diagnosis (mean ± standard deviation)
Primary analysis	Multiple sclerosis	5720	6 (4–10)	35.5 ± 8.4
	Psoriasis	26,729	6 (4–10)	37.0 ± 8.7
	Crohn’s disease	6280	6 (4–10)	34.1 ± 9.0
	Control	40,555	7 (4–10)	35.6 ± 8.4
Sensitivity analysis	Multiple sclerosis	2319	4 (1–6)	34.9 ± 8.1
	Psoriasis	13,168	4 (1–8)	36.0 ± 8.6
	Crohn’s disease	3008	4 (1–7)	33.2 ± 8.6
	Control	19,857	5 (3–8)	34.9 ± 8.2

ICD: International Classification of Diseases.

The descriptive statistics of all study cohorts used for the primary or the secondary analyses are shown including the number of distinct gynecological ICD-10 codes recorded per individual in the 5 years before first diagnosis.

The restrictions implemented for the sensitivity analysis resulted in samples sizes of 2319 women with MS; 13,168 and 3008 women with psoriasis or CD, respectively; and 19,857 women without any of these AIDs.

### Recordings rates for pregnancy-related and other gynecological ICD-10 codes

We found that 28 ICD-10 codes were recorded less frequently for women with MS (Table 2) as compared to women without any of the AIDs in the 5 years before first diagnosis, while we did not observe any ICD-10 code to be recorded more frequently. Eighteen of these 28 ICD-10 codes are related to pregnancies, of which *Supervision of normal pregnancy* (Z34) and *Supervision of high risk pregnancy* (Z35) showed the strongest negative relations to MS. We further observed that both *Encounter for contraceptive management* (Z30) and *Encounter for procreative management* (Z31) were recorded less frequently for women with MS. Three ICD-10 codes associated with disorders of the menstrual cycle as well as *Female infertility* (N97) were also associated with lower ORs of MS. Finally, four other gynecological diagnoses—*Other inflammation of vagina and vulva* (N76), *Noninflammatory disorders of ovary, fallopian tube and broad ligament* (N83), *Erosion and ectropion of cervix uteri* (N86), and *Other noninflammatory disorders of vagina* (N89) were recorded less frequently for the MS cohort.

**Table 2.** ICD-10 codes associated with lower odds ratios of MS in the primary analysis.

ICD-10 code	*N* MS	*N* Controls	OR (95% CI)	*p*-value	Adjusted *p*-value
Z30—Encounter for contraceptive management	**4347**	**33,999**	**0.59 (0.56–0.64)**	**2.05 × 10^−51^**	**1.54 × 10^−49^**
Z34—Supervision of normal pregnancy	**693**	**7127**	**0.61 (0.56–0.67)**	**1.21 × 10^−28^**	**9.12 × 10^−27^**
Z35—Supervision of high-risk pregnancy	**423**	**4258**	**0.66 (0.60–0.74)**	**3.32 × 10^−14^**	**2.49 × 10^−12^**
O09—Pregnancy duration	**453**	**4430**	**0.68 (0.61–0.75)**	**2.35 × 10^−13^**	**1.77 × 10^−11^**
Z31—Encounter for procreative management	**534**	**5024**	**0.71 (0.65–0.78)**	**2.94 × 10^−12^**	**2.20 × 10^−10^**
Z32—Encounter for pregnancy test and childbirth and childcare instruction	**503**	**4678**	**0.72 (0.66–0.80)**	**8.77 × 10^−11^**	**6.59 × 10^−09^**
Z39—Encounter for maternal postpartum care and examination	**444**	**4194**	**0.71 (0.64–0.79)**	**8.81 × 10^−11^**	**6.62 × 10^−09^**
O26—Maternal care for other conditions	**420**	**4008**	**0.70 (0.63–0.78)**	**9.32 × 10^−11^**	**7.00 × 10^−09^**
O99—Other maternal diseases	**303**	**3033**	**0.68 (0.60–0.76)**	**4.15 × 10^−10^**	**3.11 × 10^−08^**
O21—Excessive vomiting in pregnancy	**198**	**2084**	**0.65 (0.56–0.75)**	**1.12 × 10^−08^**	**8.40 × 10^−07^**
Z33—Pregnant state	**292**	**2827**	**0.70 (0.62–0.80)**	**2.98 × 10^−08^**	**2.24 × 10^−06^**
O20—Hemorrhage in early pregnancy	**251**	**2470**	**0.69 (0.61–0.79)**	**9.09 × 10^−08^**	**6.83 × 10^−06^**
O36—Maternal care for other fetal problems	**173**	**1723**	**0.69 (0.59–0.81)**	**5.44 × 10^−06^**	**4.08 × 10^−04^**
O24—Gestational diabetes	**77**	**896**	**0.60 (0.47–0.75)**	**1.57 × 10^−05^**	**1.18 × 10^−03^**
O80—Encounter for full-term uncomplicated delivery	**156**	**1548**	**0.69 (0.59–0.82)**	**2.04 × 10^−05^**	**1.53 × 10^−03^**
O92—Other disorders of breast and disorders of lactation associated with pregnancy and the puerperium	**172**	**1655**	**0.72 (0.61–0.84)**	**4.86 × 10^−05^**	**3.65 × 10^−03^**
O48—Late pregnancy	**132**	**1293**	**0.70 (0.59–0.85)**	**1.67 × 10^−04^**	**1.25 × 10^−02^**
O62—Abnormalities of forces of labor	**107**	**1082**	**0.68 (0.56–0.84)**	**2.27 × 10^−04^**	**1.70 × 10^−02^**
O71—Other obstetric trauma	**29**	**402**	**0.50 (0.34–0.73)**	**3.56 × 10^−04^**	**2.67 × 10^−02^**
O32—Maternal care for malpresentation of fetus	**120**	**1173**	**0.71 (0.59–0.86)**	**3.98 × 10^−04^**	**2.99 × 10^−02^**
N89—Other noninflammatory disorders of vagina	**3272**	**26,025**	**0.74 (0.70–0.79)**	**4.01 × 10^−25^**	**3.01 × 10^−23^**
N91—Absent, scanty, and rare menstruation	**1116**	**9671**	**0.77 (0.71–0.82)**	**8.00 × 10^−14^**	**6.00 × 10^−12^**
N92—Excessive, frequent, and irregular menstruation	**2052**	**16,358**	**0.83 (0.78–0.88)**	**1.21 × 10^−10^**	**9.05 × 10^−09^**
N83—Noninflammatory disorders of ovary, fallopian tube, and broad ligament	**649**	**5718**	**0.78 (0.72–0.85)**	**2.19 × 10^−08^**	**1.65 × 10^−06^**
N97—Female infertility	**244**	**2429**	**0.69 (0.60–0.79)**	**9.38 × 10^−08^**	**7.04 × 10^−06^**
N76—Other inflammation of vagina and vulva	**1596**	**12,669**	**0.85 (0.80–0.90)**	**2.28 × 10^−07^**	**1.72 × 10^−05^**
N94—Pain and other conditions associated with female genital organs and menstrual cycle	**1935**	**15,085**	**0.86 (0.81–0.91)**	**2.67 × 10^−07^**	**2.00 × 10^−05^**
N86—Erosion and ectropion of cervix uteri	**1232**	**9599**	**0.88 (0.82–0.94)**	**2.20 × 10^−04^**	**1.65 × 10^−02^**

ICD-10: International Classification of Diseases 10th Revision; *N*: number of women; MS: multiple sclerosis; OR: odds ratio; CI: confidence interval; adjusted *p*-value: *p*-value adjusted for multiple testing.

ICD-10 codes are ordered by relation to pregnancy or reproductive medicine (rows 1–20) and *p*-value.

Associations of ICD-10 codes with lower odds ratios of multiple sclerosis, which reach statistical significance in the comparison to controls without autoimmune disease. Statistically significant results are highlighted in bold.

To investigate to which degree the associations of the 10 gynecological ICD-10 codes unrelated to pregnancies with lower ORs of MS can be explained by a negative relation of pregnancies with MS, we performed the same analysis adjusting for pregnancy occurrences. Here, all 10 ICD-10 codes were still significantly associated with lower ORs of MS (Supplementary Table 3). However, the associations were less pronounced.

To investigate the possibility of a reversed causality between MS risk and pregnancies or gynecological disorders, we performed a sensitivity analysis excluding all women with ICD-10 codes suggestive of possible demyelinating events before diagnosis. Here, all ICD-10 codes with significant results in the primary analysis except for two pregnancy-related ICD-10 codes were still negatively associated with MS (Table 3). For all ICD-10 codes the ORs of MS were even lower as compared to the primary analysis.

**Table 3.** ICD-10 codes associated with lower odds ratios of MS in the primary analysis—sensitivity analysis.

ICD-10 code	*N* MS	*N* Controls	OR (95% CI)	*p*-value	Adjusted *p*-value
Z30—Encounter for contraceptive management	**1416**	**15,967**	**0.37 (0.34–0.41)**	**1.16 × 10^−99^**	**3.17 × 10^−98^**
Z34—Supervision of normal pregnancy	**233**	**3529**	**0.48 (0.41–0.55)**	**9.97 × 10^−24^**	**2.72 × 10^−22^**
Z35—Supervision of high-risk pregnancy	**129**	**2099**	**0.47 (0.39–0.57)**	**2.66 × 10^−15^**	**7.28 × 10^−14^**
O09—Pregnancy duration	**151**	**2187**	**0.54 (0.45–0.64)**	**1.86 × 10^−12^**	**5.09 × 10^−11^**
Z31—Encounter for procreative management	**154**	**2320**	**0.51 (0.43–0.61)**	**1.66 × 10^−14^**	**4.53 × 10^−13^**
Z32—Encounter for pregnancy test and childbirth and childcare instruction	**159**	**2183**	**0.58 (0.49–0.68)**	**1.39 × 10^−10^**	**3.80 × 10^−09^**
Z39—Encounter for maternal postpartum care and examination	**155**	**2131**	**0.56 (0.48–0.67)**	**7.97 × 10^−11^**	**2.18 × 10^−09^**
O26—Maternal care for other conditions	**131**	**1886**	**0.54 (0.45–0.65)**	**1.10 × 10^−10^**	**3.01 × 10^−09^**
O99—Other maternal diseases	**99**	**1411**	**0.56 (0.45–0.69)**	**7.47 × 10^−08^**	**2.04 × 10^−06^**
O21—Excessive vomiting in pregnancy	**52**	**911**	**0.46 (0.35–0.61)**	**8.19 × 10^−08^**	**2.24 × 10^−06^**
Z33—Pregnant state	**86**	**1269**	**0.54 (0.43–0.68)**	**9.77 × 10^−08^**	**2.67 × 10^−06^**
O20—Hemorrhage in early pregnancy	**77**	**1154**	**0.54 (0.42–0.68)**	**2.57 × 10^−07^**	**7.01 × 10^−06^**
O36—Maternal care for other fetal problems	**45**	**866**	**0.42 (0.31–0.57)**	**1.88 × 10^−08^**	**5.14 × 10^−07^**
O24—Gestational diabetes	**21**	**408**	**0.42 (0.27–0.65)**	**1.23 × 10^−04^**	**3.37 × 10^−03^**
O80—Encounter for full-term uncomplicated delivery	**49**	**770**	**0.52 (0.39–0.69)**	**1.02 × 10^−05^**	**2.80 × 10^−04^**
O92—Other disorders of breast and disorders of lactation associated with pregnancy and the puerperium	**50**	**755**	**0.54 (0.40–0.72)**	**3.16 × 10^−05^**	**8.62 × 10^−04^**
O48—Late pregnancy	**42**	**682**	**0.50 (0.37–0.69)**	**1.87 × 10^−05^**	**5.12 × 10^−04^**
O62—Abnormalities of forces of labor	**38**	**513**	**0.61 (0.44–0.85)**	**3.53 × 10^−03^**	**9.64 × 10^−02^**
O71—Other obstetric trauma	**6**	**184**	**0.27 (0.12–0.61)**	**1.57 × 10^−03^**	**4.29 × 10^−02^**
O32—Maternal care for malpresentation of fetus	**47**	**583**	**0.66 (0.49–0.90)**	**7.85 × 10^−03^**	**2.15 × 10^−01^**
N89—Other noninflammatory disorders of vagina	**1017**	**12,061**	**0.50 (0.46–0.55)**	**1.64 × 10^−54^**	**4.48 × 10^−53^**
N91—Absent, scanty, and rare menstruation	**330**	**4236**	**0.60 (0.53–0.68)**	**3.99 × 10^−16^**	**1.09 × 10^−14^**
N92—Excessive, frequent, and irregular menstruation	**597**	**7032**	**0.63 (0.57–0.70)**	**3.69 × 10^−20^**	**1.01 × 10^−18^**
N83—Noninflammatory disorders of ovary, fallopian tube, and broad ligament	**165**	**2290**	**0.58 (0.50–0.69)**	**1.51 × 10^−10^**	**4.11 × 10^−09^**
N97—Female infertility	**77**	**1079**	**0.58 (0.46–0.73)**	**5.35 × 10^−06^**	**1.46 × 10^−04^**
N76—Other inflammation of vagina and vulva	**451**	**5394**	**0.64 (0.58–0.72)**	**1.02 × 10^−15^**	**2.80 × 10^−14^**
N94—Pain and other conditions associated with female genital organs and menstrual cycle	**550**	**6388**	**0.65 (0.59–0.72)**	**1.47 × 10^−16^**	**4.03 × 10^−15^**
N86—Erosion and ectropion of cervix uteri	**380**	**4327**	**0.70 (0.62–0.79)**	**1.32 × 10^−09^**	**3.59 × 10^−08^**

ICD-10: International Classification of Diseases 10th Revision; *N*: number of women; MS: multiple sclerosis; OR: odds ratio; CI: confidence interval; adjusted *p*-value: *p*-value adjusted for multiple testing.

ICD-10 codes are ordered by relation to pregnancy or reproductive medicine (rows 1–20) and *p*-value of the association in the main analysis (Table 2).

For the sensitivity analysis, we excluded women with recordings of ICD-10 codes suggestive of a demyelinating event in the 5 years before first diagnosis. Statistically significant results are highlighted in bold.

**Figure 1.**
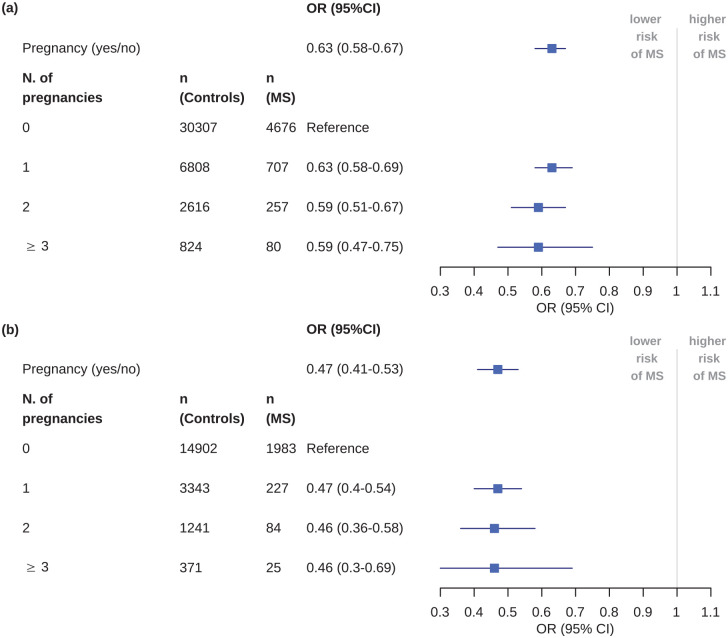
The association of pregnancies with multiple sclerosis risk depending on the number of pregnancies. Regression analyses were performed on women with evidence for one or more pregnancies in the years before first diagnosis. The analysis was performed for (a) the main cohorts and (b) the cohorts selected for the sensitivity analysis.

We further investigated a possible dose effect of pregnancies on MS diagnosis. While we could observe lower ORs of MS for women with more than one pregnancy, these differences were not significant (Figure 1).

**Figure 2.**
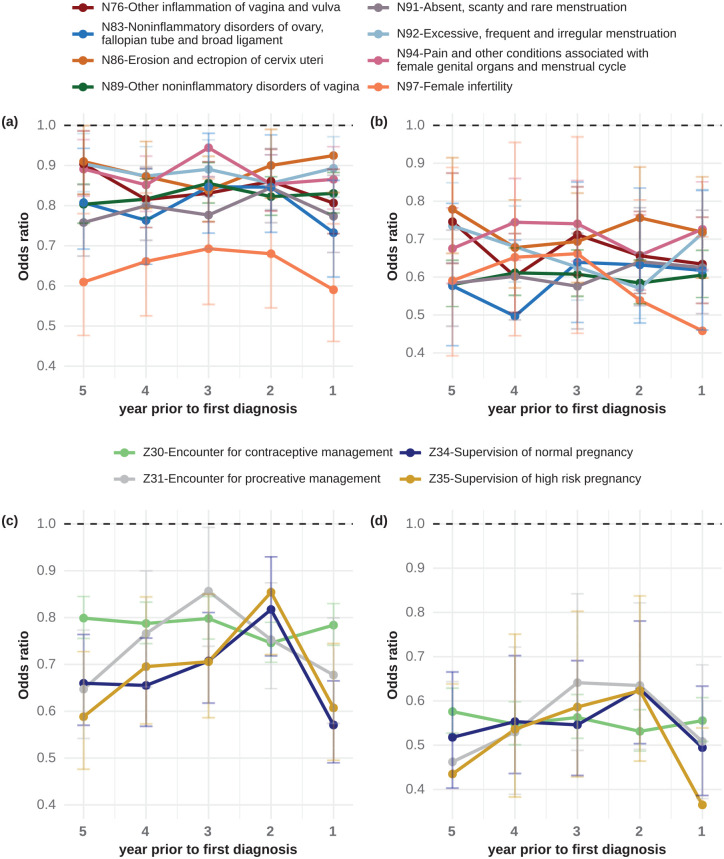
Single-year analysis on ICD-10 codes associated with lower odds ratios of multiple sclerosis. Odds ratios (ORs) of multiple sclerosis (MS) are below 1.0 for ICD-10 codes associated with lower ORs of MS for each of the 5 years before first diagnosis in the (a, c) primary analysis as well as in the (b, d) sensitivity analysis for which we removed patients with possible demyelinating events in the five years before first diagnosis. ICD-10 codes related to pregnancies or reproductive medicine are shown in c and d; other gynecological ICD-10 codes in a and b. In the sensitivity analysis (b, d), the ORs of MS were even lower as compared to the main analysis (a, c).

To investigate when the differences in recording rates first become apparent, we performed separate analyses for each of the 5 years prior to diagnosis. The ORs of all 28 ICD-10 codes were below 1.0 in all analyses, showing that even five years before diagnosis the recordings rates differ between women with MS and controls (Figure 2 for a selection of ICD-10 codes).

Next, we investigated whether the observed associations are specific for MS or shared by other AIDs by using control cohorts of women with other AIDs. In comparison to women with psoriasis, 23 of the 28 ICD-10 codes were still negatively related to MS. In comparison to the CD cohort, only two ICD-10 codes (unrelated to pregnancies) were negatively associated with MS (Supplementary Table 4).

### Gynecologist encounters in the 5 years before diagnosis

**Figure 3.**
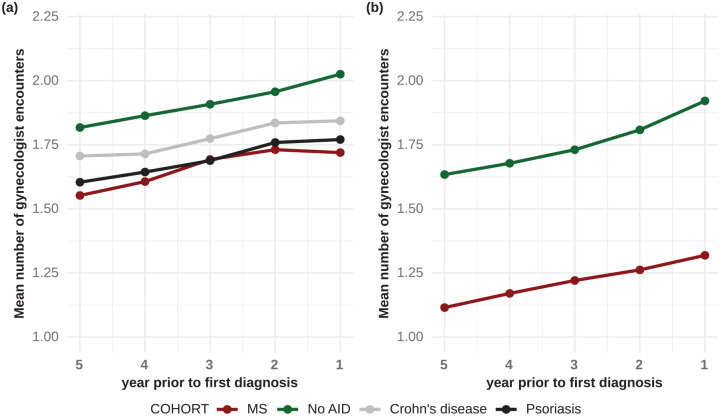
Number of gynecological encounters in the 5 years before first diagnosis. Mean number of gynecologist encounters were calculated for each of the 5 years before first diagnosis separately for the cohorts selected for the (a) primary analysis as well as for the (b) sensitivity analysis. AID: autoimmune disease; MS: multiple sclerosis.

In the 5 years before diagnosis, women with MS had fewer gynecologist encounters as compared to women without AIDs (1.66 vs 1.91 encounters per person and year, Figure 3(a)). In the cohorts selected for the sensitivity analysis, this difference was even more pronounced with 1.21 and 1.75 gynecological visits, respectively (Figure 3(b)). In a regression analysis, the number of gynecological visits were negatively associated with MS diagnosis (OR = 0.79, 95% CI = 0.77–0.81, *p* = 2.12×^10−51^). This was still observable when adjusting for the calculated number of pregnancies (OR = 0.83, 95% CI = 0.80–0.85, *p* = 2.47×^10−31^). This association was more pronounced for the sensitivity analysis cohorts (OR = 0.59, 95% CI = 0.56–0.62, *p* = 6.80×^10−89^ and OR = 0.62, 95% CI = 0.58–0.65, *p* = 6.15×^10−65^ with or without adjustment for pregnancies, respectively).

Women with MS also had fewer gynecologist encounters as compared to women with CD or psoriasis (1.66 vs 1.77 and 1.69 per person and years, respectively, Figure 3(a)). These differences were, however, less pronounced.

## Discussion

This retrospective study provides evidence that pregnancies are associated with a lower risk of MS. We observed that 18 pregnancy-related ICD-10 codes were recorded less frequently for women with MS as compared to controls. In a sensitivity analysis excluding women with evidence for possible demyelinating events before diagnosis, these associations were even more pronounced. Furthermore, the negative relation of pregnancies with disease risk was evident for all 5 years before diagnosis and did not become weaker for the years more distant to diagnosis. These results suggest that these effects precede the development of MS and are, therefore, independent of a possible reverse causality. Previous studies raised the hypothesis of the existence of a prodromal phase of MS.^17–19^ In our previous study, however, we found evidence for demyelinating events explaining the observed increased use of the healthcare system of patients with MS in the years before diagnosis.^13^ The characteristics and the duration of a hypothesized prodromal phase of MS are currently unknown. While our results suggest that the association of pregnancies and MS risk precede the disease or a phase with ongoing but undiagnosed disease, we cannot fully exclude the possibility that a prodromal phase with yet-to-be-defined clinical features might have an effect on pregnancies. Our data do, however, suggest that the observed effects are independent of or possibly in addition to a hypothesized reversed causality.

There was no clear evidence for a dose effect of pregnancies on MS risk in this study. Some previous studies found that each birth or pregnancy further decreased the risk for MS.^9,11,20^ However, this could not be confirmed in other studies.^10,21^ A possible explanation for the lack of evidence for a dose response in this study might be lack of power. In addition, as the data do not include a parameter that can directly be used to determine the number of pregnancies, our analysis might be imprecise. Furthermore, only information on pregnancies in the 5 years before diagnosis were available. However, as other studies also could not identify a dose effect of pregnancies on MS risk or age at manifestation, it can be hypothesized that the factors linking pregnancies to a reduced MS risk might not depend on the duration or the number of pregnancies. Multiple changes in DNA methylation occur during pregnancy,^22^ and if these changes were to impact the risk for MS such an effect could be expected to last for several years after a pregnancy regardless of following pregnancies.

In addition to the pregnancy-related ICD-10 codes eight other gynecological disorders were associated with lower ORs of MS including three disorders of the menstrual cycle as well as female infertility. Two previous studies did not find a negative relation between infertility and MS risk.^8,11^ We also observed lower gynecologist visit rates for women with MS as compared to controls, even when taking pregnancies into account. Fewer pregnancy-related physician encounters in women with MS in the years before diagnosis have previously been reported.^18^ Again, these associations were stronger when analyzing the sensitivity analysis cohorts. A possible explanation for these findings would be that women who are not trying to or getting pregnant are seen by gynecologists less frequently and are therefore less likely to be diagnosed with gynecological disorders. We attempted to investigate this hypothesis by adjusting for the occurrence of pregnancies and while we could observe that the mentioned non-pregnancy-related associations were weaker in this analysis, they still remained significant. While these results need replication and further investigation using more detailed clinical data, they could hint at possible relationships between hormonal changes and other gynecological disorders and protection from MS.

Finally, we observed that *Encounter for contraceptive management* (Z30) and *Encounter for procreative management* (Z31) were associated with lower ORs of MS. The lower recording rates for Z31 might suggest that women who do not seek medical advice for procreation reasons and might therefore become pregnant less frequently could be at higher risk for MS. This would support the hypothesis of a protective effect of pregnancies on MS risk. It was, however, surprising that also Z30 was negatively associated with MS risk. A possible interpretation of this finding is that women who do not try to become pregnant might obtain the needed prescriptions for contraceptives from other physicians and do not visit their gynecologists regularly. In the sensitivity analysis, the negative association of Z30 with MS was markedly more pronounced, which argues for an effect independent of a hypothesized reversed causality. Multiple previous studies investigated the association of oral contraceptives (OCs) and MS risk with conflicting results.^20,23–25^ These previous studies were based on the analysis of relatively small cohorts of just a few hundred women with MS or clinically isolated syndrome (CIS). Further studies with larger cohorts with available clinical and drug prescription data are needed to shed light on the association between contraception and MS risk.

While most of the observed relations of gynecological ICD-10 codes with MS risk could be confirmed in comparison to the cohort of women with psoriasis, only two (not pregnancy-related) ICD-10 codes showed an association with MS in comparison to women with CD. Pregnancies have not been shown to have a consistent effect on the disease course of CD or psoriasis.^26–29^ A number of studies have shown that genetic risk loci are shared between different AIDs, suggesting—at least to some degree—shared pathophysiological mechanisms.^30,31^ Our data suggest that the association of pregnancies and possibly different gynecological disorders with disease risk might be shared by some AIDs but not by others. Shared genetic liability and shared pathomechanisms between AIDs might be a possible explanation for these findings.

### Limitations

The ICD-10 codes are not audited and reflect the coding practices of German physicians. Hospital claims are not covered. The data do not include a direct parameter for pregnancies and the occurrences and number of pregnancies were estimated using recorded pregnancy-related ICD-10 codes. Furthermore, there is no reliable information available on which pregnancies were full term and led to childbirth or on their duration, which made an assessment of the effects of pregnancy duration or outcome impossible. Assuming that a relevant portion of the recorded pregnancies were not full term, our estimation of the effect of pregnancies on MS risk might be biased. In addition, we could not adjust for known or suspected MS risk factors such as smoking, vitamin D deficiency, or obesity. A further limitation is the potential of confounding that might be induced by the non-experimental study design. The BASHIP data cover approximately 85% of the Bavarian general population,^32^ resulting in a high degree of generalizability. The 15% not covered are persons with private health insurance including civil servants, the self-employed, and those earning above a set income threshold. While these factors could theoretically have an impact on the studied ICD-10 codes, this could not be assessed in this study.

## Conclusion

Our results suggest a possible protective effect of pregnancies on MS risk. With an increase of the maternal age at first childbirth^33,34^ and decreasing birth rates^35^ in the last decades, a protective effect of pregnancies on disease risk could, at least in part, explain the increasing gender gap in MS incidence. We also observed previously not reported associations of gynecological disorders unrelated to pregnancies with lower MS risk. Whether these observations are explained by the observed lower gynecologist encounters rates in women with MS in the years before first diagnosis or whether they represent truly independent associations of gynecological disorders and MS risk, needs further investigation. The observed associations might, to some degree, be shared by different AIDs.

## Supplemental Material

sj-docx-1-msj-10.1177_13524585221080542 – Supplemental material for Association of pregnancies with risk of multiple sclerosis Click here for additional data file. Supplemental material, sj-docx-1-msj-10.1177_13524585221080542 for Association of pregnancies with risk of multiple sclerosis by Christiane Gasperi, Alexander Hapfelmeier, Antonius Schneider, Klaus A Kuhn, Ewan Donnachie and Bernhard Hemmer in Multiple Sclerosis Journal
