# Supplementary material for: Association of pregnancies with risk of multiple sclerosis
Source: Mult Scler. 2022 Mar 18;28(10):1630–40. doi: 10.1177/13524585221080542 (PMC9315178; doi:10.1177/13524585221080542)
Supplement: sj-docx-1-msj-10.1177_13524585221080542 – Supplemental material for Association of pregnancies with risk of multiple sclerosis [file sj-docx-1-msj-10.1177_13524585221080542.docx]

Supplementary material for „Association of pregnancies with risk of multiple sclerosis”

Christiane Gasperi, Alexander Hapfelmeier, Klaus A. Kuhn, Ewan Donnachie and
 Bernhard Hemmer

[Supplementary Table 1. List of ICD-10 codes used for cohort restrictions 2](#_Toc84690797)

[Supplementary Table 2. List of analyzed ICD-10 codes 3](#_Toc84690798)

[Supplementary Table 3. ICD-10 codes associated with lower odds ratios of multiple sclerosis in the primary analysis – analysis with adjustment for pregnancies. 5](#_Toc84690799)

[Supplementary Table 4. ICD-10 codes associated with lower odds ratios of multiple sclerosis in the primary analysis in comparison to women with Crohn’s disease or psoriasis. 6](#_Toc84690800)

# Supplementary Table 1. List of ICD-10 codes used for cohort restrictions

| **Restriction** | **ICD-10 code / group** | **Description** |
| --- | --- | --- |
| Exclusion of patients with other possible demyelinating or inflammatory diseases of the central nervous system | G04 | Encephalitis, myelitis and encephalomyelitis |
|  | G05 | Encephalitis, myelitis and encephalomyelitis in diseases classified elsewhere |
|  | G09 | Sequelae of inflammatory diseases of central nervous system |
|  | G36 | Other acute disseminated demyelination |
|  | G37 | Other demyelinating disease of central nervous system |
|  | H46 | Optic neuritis |
|  | H47 | Other disorders of optic [2^nd^] nerve and visual pathways |
|  | H48 | Disorders of optic [2nd] nerve and visual pathways in diseases classified elsewhere |
|  | R90 | Abnormal findings on diagnostic imaging of central nervous system |
| Sensitivity analysis: Exclusion of patients with possible unrecognized demyelinating events before first diagnosis | G00-G99 | Diseases of the nervous system |
|  | I60-I69 | Cerebrovascular diseases |
|  | H53 | Visual disturbances |
|  | H81 | Disorders of vestibular function |
|  | N31 | Neuromuscular dysfunction of bladder, not elsewhere classified |
|  | R20 | Disturbances of skin sensation |
|  | R26 | Abnormalities of gait and mobility |
|  | R32 | Unspecified urinary incontinence |
|  | R39 | Other and unspecified symptoms and signs involving the urinary system |
|  | R42 | Dizziness and giddiness |
|  | R47 | Speech disturbances, not elsewhere classified |

# Supplementary Table 2. List of analyzed ICD-10 codes

| **ICD** | **Description** |
| --- | --- |
| D06 | Carcinoma in situ of cervix uteri |
| D25 | Leiomyoma of uterus |
| D26 | Other benign neoplasms of uterus |
| D27 | Benign neoplasm of ovary |
| D39 | Neoplasm of uncertain behavior of female genital organs |
| E28 | Primary ovarian failure |
| N70 | Salpingitis and oophoritis |
| N71 | Inflammatory disease of uterus, except cervix |
| N72 | Inflammatory disease of cervix uteri |
| N73 | Other female pelvic inflammatory diseases |
| N75 | Diseases of Bartholin's gland |
| N76 | Other inflammation of vagina and vulva |
| N77 | Vulvovaginal ulceration and inflammation in diseases classified elsewhere |
| N80 | Endometriosis |
| N81 | Female genital prolapse |
| N83 | Noninflammatory disorders of ovary, fallopian tube and broad ligament |
| N84 | Polyp of female genital tract |
| N85 | Other noninflammatory disorders of uterus, except cervix |
| N86 | Erosion and ectropion of cervix uteri |
| N87 | Dysplasia of cervix uteri |
| N88 | Other noninflammatory disorders of cervix uteri |
| N89 | Other noninflammatory disorders of vagina |
| N91 | Absent, scanty and rare menstruation |
| N92 | Excessive, frequent and irregular Menstruation |
| N93 | Other abnormal uterine and vaginal bleeding |
| N94 | Pain and other conditions associated with female genital organs and menstrual cycle |
| N95 | Menopausal and other perimenopausal disorders |
| N97 | Female infertility |
| O00 | Ectopic pregnancy |
| O02 | Other abnormal products of conception |
| O03 | Spontaneous abortion |
| O04 | Complications following (induced) termination of pregnancy |
| O06 | Unspecified abortion |
| O09 | Pregnancy duration |
| O12 | Gestational [pregnancy-induced] edema and proteinuria without hypertension |
| O14 | Pre-eclampsia |
| O20 | Hemorrhage in early pregnancy |
| O21 | Excessive vomiting in pregnancy |
| O22 | Venous complications and hemorrhoids in pregnancy |
| O23 | Infections of genitourinary tract in pregnancy |
| O24 | Gestational diabetes |
| O26 | Maternal care for other conditions |
| O28 | Abnormal findings on antenatal screening of mother |
| O30 | Multiple gestation |
| O32 | Maternal care for malpresentation of fetus |
| O33 | Maternal care for disproportion |
| O34 | Maternal care for abnormality of pelvic organs |
| O35 | Maternal care for known or suspected fetal abnormality and damage |
| O36 | Maternal care for other fetal problems |
| O41 | Other disorders of amniotic fluid and membranes |
| O42 | Premature rupture of membranes |
| O43 | Placental disorders |
| O44 | Placenta previa |
| O46 | Antepartum hemorrhage, not elsewhere classified |
| O47 | False labor |
| O48 | Late pregnancy |
| O60 | Preterm labor |
| O62 | Abnormalities of forces of labor |
| O68 | Labor and delivery complicated by abnormality of fetal acid-base balance |
| O71 | Other obstetric trauma |
| O80 | Encounter for full-term uncomplicated delivery |
| O82 | Encounter for cesarean delivery without indication |
| O90 | Complications of the puerperium, not elsewhere classified |
| O91 | Infections of breast associated with pregnancy, the puerperium and lactation |
| O92 | Other disorders of breast and disorders of lactation associated with pregnancy and the puerperium |
| O99 | Other maternal diseases |
| Q51 | Congenital malformations of uterus and cervix |
| R87 | Abnormal findings in specimens from female genital organs |
| Z30 | Encounter for contraceptive management |
| Z31 | Encounter for procreative management |
| Z32 | Encounter for pregnancy test and childbirth and childcare instruction |
| Z33 | Pregnant state |
| Z34 | Supervision of normal pregnancy |
| Z35 | Supervision of high risk pregnancy |
| Z36 | Encounter for antenatal screening of mother |
| Z37 | Outcome of delivery |
| Z39 | Encounter for maternal postpartum care and examination |

# Supplementary Table 3. ICD-10 codes associated with lower odds ratios of multiple sclerosis in the primary analysis – analysis with adjustment for pregnancies.

| **ICD-10 code** | **N MS** | **N Controls** | ***OR* (95% *CI*)** | ***p*-value** | **adjusted *p*-value** |
| --- | --- | --- | --- | --- | --- |
| N76 - Other inflammation of vagina and vulva | **1,596** | **12,669** | **0.89 (0.83-0.94)** | **1.38×10^-04^** | **1.34×10^-03^** |
| N83 - Noninflammatory disorders of ovary, fallopian tube and broad ligament | **649** | **5,718** | **0.82 (0.75-0.89)** | **4.31×10^-06^** | **4.21×10^-05^** |
| N86 - Erosion and ectropion of cervix uteri | **1232** | **9599** | **0.90 (0.84-0.96)** | **2.74×10^-03^** | **2.67×10^-02^** |
| N89 - Other noninflammatory disorders of vagina | **3,272** | **26,025** | **0.78 (0.74-0.83)** | **7.41×10^-17^** | **7.24×10^-16^** |
| N91 - Absent, scanty and rare menstruation | **1,116** | **9671** | **0.86 (0.80-0.93)** | **7.02×10^-05^** | **6.86×10^-04^** |
| N92 - Excessive, frequent and irregular menstruation | **2,052** | **16,358** | **0.86 (0.81-0.91)** | **1.29×10^-07^** | **1.26×10^-06^** |
| N94 - Pain and other conditions associated with female genital organs and menstrual cycle | **1,935** | **15,085** | **0.87 (0.82-0.92)** | **2.74×10^-06^** | **2.67×10^-05^** |
| N97 - Female infertility | **244** | **2,429** | **0.78 (0.68-0.89)** | **3.03×10^-04^** | **2.96×10^-03^** |
| Z30 - Encounter for contraceptive management | **4,347** | **33,999** | **0.64 (0.59-0.68)** | **1.29×10^-38^** | **1.26×10^-37^** |
| Z31 - Encounter for procreative management | **534** | **5,024** | **0.82 (0.75-0.91)** | **1.22×10^-04^** | **1.19×10^-03^** |

Associations of ICD-10 codes with lower odds ratios of multiple sclerosis which reached statistical significance in the main analysis were analysed in a secondary regression analysis adjusted for the occurrence of pregnancies. Statistically significant results are highlighted in bold.

Abbreviations: CI = Confidence interval; MS = multiple sclerosis; N = number of women; OR = Odds Ratio, adjusted *p*-value = *p*-value adjusted for multiple testing.

# Supplementary Table 4. ICD-10 codes associated with lower odds ratios of multiple sclerosis in the primary analysis in comparison to women with Crohn’s disease or psoriasis.

| **ICD code** | **N**  **MS** | **vs. Psoriasis** | | | | **vs. Crohn’s disease** | | | |
| --- | --- | --- | --- | --- | --- | --- | --- | --- | --- |
|  |  | **N** | **OR (95% CI)** | ***p*-value** | **adjusted *p*** | **N** | **OR (95% CI)** | ***p*-value** | **adjusted *p*** |
| N76 - Other inflammation of vagina and vulva | 1,596 | **8,130** | **0.87 (0.82-0.93)** | **3.44×10^-05^** | **2.58×10^-03^** | **1,978** | **0.85 (0.78-0.92)** | **3.43×10^-05^** | **2.57×10^-02^** |
| N83 - Noninflammatory disorders of ovary, fallopian tube and broad ligament | 649 | **3,641** | **0.84 (0.77-0.92)** | **1.20×10^-04^** | **8.98×10^-03^** | **915** | **0.71 (0.64-0.79)** | **9.29×10^-10^** | **6.98×10^-08^** |
| N86 - Erosion and ectropion of cervix uteri | 1,232 | 5,554 | 1.01 (0.94-1.09) | 7.12×10^-01^ | 1.00×10^-00^ | 1,404 | 0.99 (0.91-1.08) | 8.21×10^-01^ | 1.00×10^-00^ |
| N89 - Other noninflammatory disorders of vagina | 3,272 | 15,520 | 0.94 (0.88-0.99) | 2.97×10^-02^ | 1.00×10^-00^ | 3,799 | 0.89 (0.82-0.95) | 1.34×10^-03^ | 1.01×10^-01^ |
| N91 - Absent, scanty and rare menstruation | 1,116 | **6,077** | **0.78 (0.73-0.84)** | **3.08×10^-11^** | **2.31×10^-09^** | 1,325 | 0.92 (0.84-1.01) | 7.26×10^-02^ | 1.00×10^-00^ |
| N92 - Excessive, frequent and irregular menstruation | 2,052 | 9,783 | 0.97 (0.92-1.03) | 3.47×10^-01^ | 1.00×10^-00^ | 2,438 | 0.90 (0.83-0.97) | 5.19×10^-03^ | 3.89×10^-01^ |
| N94 - Pain and other conditions associated with female genital organs and menstrual cycle | 1,935 | 8,700 | 1.01 (0.95-1.07) | 7.36×10^-01^ | 1.00×10^-00^ | 2,410 | 0.88 (0.82-0.95) | 1.20×10^-03^ | 9.03×10^-02^ |
| N97 - Female infertility | 244 | **1,480** | **0.72 (0.63-0.83)** | **3.93×10^-06^** | **2.95×10^-04^** | 278 | 0.89 (0.74-1.06) | 1.83×10^-01^ | 1.00×10^-00^ |
| O09 - Pregnancy duration | 453 | **2,625** | **0.70 (0.63-0.78)** | **4.16×10^-11^** | **3.13×10^-09^** | 437 | 1.11 (0.96-1.27) | 1.47×10^-01^ | 1.00×10^-00^ |
| O20 - Hemorrhage in early pregnancy | 251 | **1,589** | **0.65 (0.57-0.74)** | **7.65×10^-10^** | **5.74×10^-08^** | 265 | 0.99 (0.83-1.18) | 8.93×10^-01^ | 1.00×10^-00^ |
| O21 - Excessive vomiting in pregnancy | 198 | **1,270** | **0.63 (0.54-0.74)** | **6.82×10^-09^** | **5.12×10^-07^** | 232 | 0.92 (0.76-1.12) | 3.94×10^-01^ | 1.00×10^-00^ |
| O24 - Gestational diabetes | 77 | **691** | **0.48 (0.38-0.60)** | **1.07×10^-09^** | **8.03×10^-08^** | 87 | 0.89 (0.66-1.22) | 4.79×10^-01^ | 1.00×10^-00^ |
| O26 - Maternal care for other conditions | 420 | **2,595** | **0.65 (0.58-0.73)** | **1.88×10^-14^** | **1.41×10^-12^** | 428 | 1.04 (0.90-1.20) | 6.12×10^-01^ | 1.00×10^-00^ |
| O32 - Maternal care for malpresentation of fetus | 120 | **771** | **0.65 (0.54-0.79)** | **1.86×10^-05^** | **1.40×10^-03^** | 108 | 1.13 (0.87-1.48) | 3.57×10^-01^ | 1.00×10^-00^ |
| O36 - Maternal care for other fetal problems | 173 | **1,209** | **0.59 (0.50-0.70)** | **3.54×10^-10^** | **2.66×10^-08^** | 178 | 1.02 (0.82-1.26) | 8.65×10^-01^ | 1.00×10^-00^ |
| O48 - Late pregnancy | 132 | **941** | **0.58 (0.48-0.70)** | **8.45×10^-09^** | **6.35×10^-07^** | 122 | 1.12 (0.87-1.44) | 3.87×10^-01^ | 1.00×10^-00^ |
| O62 - Abnormalities of forces of labor | 107 | **785** | **0.56 (0.46-0.69)** | **3.57×10^-09^** | **2.68×10^-06^** | 110 | 1.02 (0.78-1.34) | 8.69×10^-01^ | 1.00×10^-00^ |
| O71 - Other obstetric trauma | 29 | **238** | **0.51 (0.35-0.75)** | **6.30×10^-04^** | **4.73×10^-02^** | 45 | 0.65 (0.41-1.04) | 7.15×10^-02^ | 1.00×10^-00^ |
| O80 - Encounter for full-term uncomplicated delivery | 156 | **1,020** | **0.63 (0.53-0.75)** | **1.94×10^-07^** | **1.46×10^-05^** | 173 | 0.92 (0.74-1.15) | 4.88×10^-01^ | 1.00×10^-00^ |
| O92 - Other disorders of breast and disorders of lactation associated with pregnancy and the puerperium | 172 | **1,138** | **0.63 (0.54-0.75)** | **4.71×10^-08^** | **3.54×10^-06^** | 187 | 0.96 (0.77-1.18) | 6.73×10^-01^ | 1.00×10^-00^ |
| O99 - Other maternal diseases | 303 | **1,943** | **0.64 (0.56-0.72)** | **1.75×10^-12^** | **1.31×10^-10^** | 313 | 1.03 (0.87-1.21) | 7.19 ×10^-01^ | 1.00×10^-00^ |
| Z30 - Encounter for contraceptive management | 4,347 | 20,139 | 0.97 (0.90-1.04) | 3.42×10^-01^ | 1.00×10^-00^ | 4,906 | 0.93 (0.85-1.02) | 1.15×10^-01^ | 1.00×10^-00^ |
| Z31 - Encounter for procreative management | 534 | **2,838** | **0.80 (0.72-0.88)** | **7.69×10^-06^** | **5.77×10^-04^** | 566 | 0.98 (0.86-1.11) | 7.09×10^-01^ | 1.00×10^-00^ |
| Z32 - Encounter for pregnancy test and childbirth and childcare instruction | 503 | **2,808** | **0.74 (0.66-0.81)** | **2.78×10^-09^** | **2.09×10^-07^** | 496 | 1.12 (0.98-1.28) | 9.36×10^-02^ | 1.00×10^-00^ |
| Z33 - Pregnant state | 292 | **1,908** | **0.62 (0.55-0.70)** | **2.93×10^-13^** | **2.20×10^-11^** | 327 | 0.93 (0.79-1.10) | 4.06×10^-01^ | 1.00×10^-00^ |
| Z34 - Supervision of normal pregnancy | 693 | **4,462** | **0.59 (0.54-0.64)** | **1.14×10^-31^** | **8.56×10^-30^** | 700 | 1.05 (0.93-1.18) | 4.15×10^-01^ | 1.00×10^-00^ |
| Z35 - Supervision of high risk pregnancy | 423 | **2,744** | **0.63 (0.57-0.70)** | **7.82×10^-17^** | **5.87×10^-15^** | 422 | 1.04 (0.90-1.20) | 6.13×10^-01^ | 1.00×10^-00^ |
| Z39 - Encounter for maternal postpartum care and examination | 444 | **2,810** | **0.63 (0.57-0.70)** | **2.93×10^-17^** | **2.20×10^-15^** | 436 | 1.06 (0.92-1.22) | 4.36×10^-01^ | 1.00×10^-00^ |

Statistically significant results are highlighted in bold. Abbreviations: CI = Confidence interval; N = number of women; OR = Odds Ratio, adjusted *p*-value = *p*-value adjusted for multiple testing.
